# Obstructive sleep apnoea as a neuromuscular respiratory disease arising from an excess of central GABAergic neurotransmitters: a new disease model

**DOI:** 10.3389/fncel.2024.1429570

**Published:** 2025-01-06

**Authors:** Domenico Maurizio Toraldo, Alessandra Palma Modoni, Egeria Scoditti, Francesco De Nuccio

**Affiliations:** ^1^Respiratory Care Unit, Rehabilitation Department, “V. Fazzi” Hospital, Azienda Sanitaria Locale, San Cesario, Lecce, Italy; ^2^National Research Council (CNR), Institute of Clinical Physiology (IFC), Lecce, Italy; ^3^Laboratory of Human Anatomy, Department of Experimental Medicine, University of the Salento, Lecce, Italy

**Keywords:** obstructive sleep apnoea (OSA), microbiota, cardiovascular disease (CVD), brain-gut-microbiome axis (BGMA), ascending reticular activating system (ARAS), mesencephalic trigeminal nucleus (MTN), hypothalamic neurons of the preoptic area (POA), inhibitory neurotransmitters

## Introduction

OSA is a heterogeneous disease with variable physio-pathological and clinical manifestations, and it is associated with numerous co-morbidities (Malhotra et al., 2020). It is a chronic inflammatory disease with a low degree of activity (Toraldo et al., 2013), in which the microbiome is now widely considered to play a role. In the last few decades, research into the microbiome has rapidly evolved and has become a hot topic in basic research, both pre-clinical and clinical. Some recent studies have demonstrated that the gut microbiota (GM), situated in the human gastrointestinal tract and consisting of bacteria, viruses, fungi and protozoa, serves important functions regarding the regulation of the immune and metabolic systems and cerebral physiopathology. It is an example of a dynamic complex system, connected to the organism on a cellular, metabolic, immune and nervous level, a sophisticated network regulated by delicate internal and external equilibria (Collins et al., 2012; Cryan et al., 2019).

The gut microbiota (GM) is composed of microbial symbionts, commensal or mutualistic but also potentially harmful (i.e., pathobionts), whose equilibrium (homeostasis) is crucial to the modulation of various functions and the etiology of numerous diseases (Gill et al., 2006). Two bacterial phyla, Bacteroidetes and Firmicutes, account for 90% of the groups present in the human intestine and are essential for the maintenance of intestinal homeostasis (Cryan and Dinan, 2012; Dinan and Cryan, 2017a). A growing scientific literature supports the existence of bidirectional interaction that unfolds via hormonal, neural and immunological pathways between the brain and the intestine, indicating that this relationship plays a fundamental role in modulating cerebral physiopathology via neuronal development and control of synaptic plasticity (Human Microbiome Project Consortium, 2012; Morais et al., 2021; Romijn et al., 2008; Belkaid and Hand, 2014). Dysbiosis of the microbiota entails alteration of either the bacterial composition, with a reduction in bacterial diversity, or a proliferation of pathological bacteria that triggers the release of pro-inflammatory cytokines. Dysbiosis entails the release of pathological bacterial lipopolysaccharides (LPSs), called PAMPs (Pathogen Associated Molecular Patterns). PAMPs reduce the gene expression of proteins associated with the “tight junctions” of the intestinal epithelium (zonulin-1, occludin, claudin) via the activation of the nuclear factor NF-κB, while the pro-inflammatory cytokines IL-1β, IL-6, and TNF-α are responsible for “minimal persistent inflammation” (Levy et al., 2017; Weiss and Hennet, 2017; Stefano et al., 2018). Experimental studies of model rats have shown that intestinal dysbiosis is implicated in the physio-pathological mechanisms of OSA (Zhang et al., 2022; Badran et al., 2023; Moreno-Indias et al., 2016). Dysbiosis alters the synthesis and degradation of neurotransmitters and the regulation of entero-endocrine signaling pathways that communicate with the central nervous system (CNS) via neurotransmission (Ko et al., 2019a; Zeng et al., 2020).

Here we summarize the possible molecular mechanisms underlying OSA-microbiome interactions and discuss how various factors interact with gut dysbiosis to influence OSA. The physio-pathological mechanisms underlying OSA are intermittent nocturnal hypoxia (IH) and sleep fragmentation (SF), which can induce dysbiosis of the gut microbiota (GM), compromise the intestinal barrier, alter intestinal metabolites and generate neuroinflammation (Kheirandish-Gozal and Gozal, 2019; Tang et al., 2022; Ko et al., 2019b). These mechanisms, once activated, lead to secondary cellular oxidative stress, sympathetic activation and systemic inflammation (Moreno-Indias et al., 2015; Farre et al., 2018; Poroyko et al., 2016; Lavie, 2015).

## Discussion

### The effects of the microbiota on the brain

There is abundant scientific evidence that on the intestinal level, thanks to their ability to produce molecules and neurotransmitters, bacteria can act directly on the CNS via the vagus nerve, the neuroendocrine system and bacterial metabolites (Cryan and Dinan, 2012; Foster et al., 2017). Dinan et al. demonstrated in animal models that the GM can influence the physiology of the brain by regulating neurotransmission and synaptogenesis. In their study, they characterized the neurobiochemical profile of the forebrains of mice during three key phases of postnatal development, which coincide with the formation of the gut microbiota. They demonstrated that the molecules derived from intestinal microbes are able to cross the blood-brain barrier (BBB) and that the intestinal microbiome can thus influence cerebral neurodevelopmental trajectories (Dinan and Cryan, 2017b; Giuffrè et al., 2020). A recent review and meta-analysis observed increased levels of biomarkers of intestinal barrier dysfunction in patients with OSA, and found that these markers correlate with polysomnographic parameters indicating the seriousness of OSA (Mashaqi et al., 2023).

Previous experimental studies performed in the laboratory using rodent-based OSA models had already suggested that the absorption and barrier functions that regulate the intestinal epithelium are sensitive to the intensity of intermittent hypoxia (IH) and that the depth and intensity of IH can directly compromise the integrity of the intestinal epithelium, thereby altering “tight junctions” and increasing intestinal permeability and the inflammatory process (Xu et al., 1999; Taylor and Colgan, 2007).

The systemic inflammatory mechanisms generated by intestinal dysbiosis that determine the neuroinflammatory response are described below (de Oliveira et al., 2021; Gasmi et al., 2021; Di Tommaso et al., 2021). In detail, signal-ligands released by intestinal gram-negative bacteria during dysbiosis generate molecular structures such as lipopolysaccharides, called Pathogen-Associated Molecular Patterns (PAMPs). PAMPs bind to Pattern Recognition Receptors **(**PRRs) including Toll-Like Receptors (TLRs), which are found on Antigen-Presenting Cells **(**APCs**)** such as dendritic cells, macrophages, and T lymphocytes. This bond between PAMPs and PRRs activates the APCs, which are involved in the epigenetic, immunological and metabolic reprogramming of the entire organism. At the same time, this activation of receptors is responsible for the release of inflammatory cytokines that reduce the gene expression of proteins associated with the tight junctions of the intestinal epithelium (Danyang and Minghua, 2021; Kawai and Akira, 2010). This in turn increases intestinal permeability (involving zonulin-1, occluding, and claudin) and the release of the pro-inflammatory cytokines (IL-1β, IL-6, and TNF-α) responsible for systemic inflammation (Montagnani et al., 2023). In addition to the PAMP/TLR4 signaling pathways, PAMP**s** can activate another inflammatory mechanism by binding to dendritic cells (DCs**)**, which produce the interleukins IL-1 and IL-18. These can generate biologically active molecules such as the inflammasome NLRP3. Inflammasomes modulate a multitude of signals causing chronic pro-inflammatory responses (Chen et al., 2023; Liu et al., 2016; Blevins et al., 2022). Experimental studies conducted in the laboratory on animal models have demonstrated the role of nocturnal intermittent hypoxia (IH) and sleep fragmentation (SF) in causing intestinal dysbiosis, which in turn can intensify the development of the physio-pathological mechanisms of OSA and cause cardiorespiratory and metabolic co-morbidity (O'Connor et al., 2020; Ko et al., 2019a; Wang et al., 2024).

### The bio-molecular pathways of the ENS and the brain

Intestinal bacteria produce mainly gamma acid aminobutyric acid (GABA), dopamine (DA), norepinephrine (NE), serotonin (5-HT), and histamine in order to communicate with the enteric nervous system (ENS) (Strandwitz, 2018), but also intermediate compounds such as short-chain fatty acids (SCFAs) (Van De Wouw et al., 2018), tryptophan (Agus et al., 2018), and secondary bile acids (MahmoudianDehkordi et al., 2019). The signals generated by these neurotransmitters and molecules are transported to the brain via the fibers of the vagus nerve (VN). In response, the brain sends return signals to the enterochromaffin cells (ECCs) in the intestinal wall and to the immune system of the intestinal mucosae, again via the fibers of the vagus nerve (Baj et al., 2019). The activation of the VN improves the integrity of the intestinal wall, reduces peripheral inflammation and inhibits the release of pro-inflammatory cytokines such as IL-1β, IL-6 and TNF-α (Tracey and Chavan, 2018). The signals generated by the hypothalamus reach the pituitary and adrenal glands and communicate with the ECCs via the hypothalamic–pituitary–adrenal axis (HPA) (Nicholson et al., 2012). The complex control of entero-endocrine signaling and immune responses maintains the gut microbiota in a state of equilibrium. Although the vagus nerve is in contact with all the layers of the intestinal wall, its fibers do not cross the intestinal wall itself and thus are not in direct contact with the gut microbiota (Bonaz et al., 2018). The signals reach the microbiota via neurons ranging in number between 100 and 500 million belonging to the enteric nervous system (ENS). Although the ENS is associated with the VN, it functions independently from it. Recent laboratory studies have demonstrated that the ENS is dynamic and continuously changing (Rao and Gershon, 2017). The neurons of the ENS represent the biggest nervous system in the human body.

The neurons connected to the gastrointestinal tract (GIT) possess various chemical and mechanosensitive receptors that interact with regulatory hormones and peptides released by enterochromaffin cells (ECCs), also known as Kulchitsky cells. Although these cells represent just 1% of the epithelial cells of the GIT, they play an important role in maintaining the homeostasis of the GIT (Gwak and Chang, 2021). To date, 10 different types of ECCs have been characterized. The receptors on these cells are expressed by enteric neurons, but also by the fibers of the vagus nerve, the brainstem and the hypothalamus (DeSilva and Bloom, 2012; Richards et al., 2014).

The ENS, also called the “*brain within the gut*” or “*second brain*” (Goldstein et al., 2013), is composed of the myenteric plexus and inner submucosal plexus. It is structurally similar to the brain and operates on the basis of a similar “chemical platform” (Furness et al., 2014). The modulation and development of the neurons of the ENS is controlled by the gut microbiota. The embryogenic development of enteric neurons is based on the presence of microbial cells, as shown in studies conducted on mice (De Vadder et al., 2018).

The role played by the gut microbiota in association with acetylcholine-type neurotransmitters and neuro-regulatory peptides has been highlighted in recent research conducted on animal models. It has been seen that the secretion of Ach can be stimulated by some species of Lactobacillus (Ohgi et al., 2012; Qiu et al., 2017). Lactobacillus rhamnosus jB-1 changes the expression of GABA A receptors in the brain, and this reduces anxiety and depression (Chang et al., 2020).

Numerous articles demonstrating the production of GABA by the gut microbiota, especially Lactobacillus, Bifidobacterium, and Bacteroides, have been published (Strandwitz et al., 2019; Dover and Halpern, 1972; Quillin et al., 2021; Chen and Trombley, 1995; Jiang et al., 2015). It has been observed that treatment with Lactobacillus increases GABA levels in the hippocampus and the prefrontal cortex (Janik et al., 2016). Acetate, propionate and butyrate are the most abundant SCFAs in the human body. They are produced by the microbiota in the colon and are transferred across the blood-brain barrier (BBB) to the hypothalamus and enter the neuroglial pathways associated with GABA. In addition, SCFAs can modulate neuroinflammation and influence the immune system by regulating the differentiation, recruitment and activation of immune cells including neutrophils, macrophages, and T-cells. They do this via the GPR-41 receptor situated in the ENS, which is able to transfer the signal induced by SCFAs directly to the CNS (Frost et al., 2014). These studies clearly indicate that SCFAs produced by the microbiota play a fundamental role in cerebral processes. Other bacteria of the gut microbiota, such as Parabacteroides, can play a role in the regulation of GABA by altering the GABA: glutamate ratio and by increasing levels of glutamate in the brain. Glutamate is a natural aminoacidic that acts as an excitatory neurotransmitter in the nervous system. It is involved in cognitive functions such as learning and memory, as well as in the control of motor functions and mood (Olson et al., 2018). Dopamine and norepinephrine are directly produced by some gut bacteria such as Bacillus and Serratia, and they directly perform their excitatory functions in the CNS (Tsavkelova et al., 2000).

Some bacterial strains can independently stimulate, by means of their metabolites, the synthesis and release of neurotransmitters by ECCs. The precursors of neurotransmitters synthesized by bacteria and ECCs can enter the bloodstream, cross the blood-brain barrier and participate in the neurotransmitter synthesis cycle in the brain. The ECCs that synapse with vagal neurons are called “neuropod cells” (Dicks, 2022). Located in the intestinal epithelium, these cells synthesize and release neurotransmitters such as glutamate, which can transmit sensory signals to the brain within milliseconds via the vagus nerve. However, the mechanisms by which intestinal dysbiosis alters central neurotransmission and how these interactions influence brain functioning in OSA are not yet fully understood (Chen et al., 2021). A recent paper by Brödel et al. (2024) showed that bacteria can be modified directly in the gut using phage as non-replicative DNA vectors. Although the study was performed on mice, it opens up new prospects for microbiome-targeted therapies.

GABA neurotransmitters (Bak et al., 2006; Hertz, 2006; Struzyńska and Sulkowski, 2004) play an important role in the inhibition of the Central Nervous System (CNS), mediated by the Preoptic Area (POA), especially by slowing central nervous activity. This inhibitory function of GABA serves to balance the excitatory activity of other central neurotransmitters, such as acetylcholine, dopamine and norepinephrine, and disturbance of this balance, mainly nocturnal, contributes to a lack of tone in the hypopharyngeal and hypoglossal muscles and the diaphragm. Fundamentally, GABA helps to maintain the proper balance between excitation and inhibition of the Central Nervous System. A slight increase in inhibition reduces anxiety and its associated symptoms and can restrain potential convulsions. Further alteration of the inhibition process can induce unconsciousness when under general anesthetic and/or hypotonia (Masiulis et al., 2019). The disruption of this delicate inhibitory equilibrium can reduce muscle tone in the respiratory tract, with sedative, relaxant and hypnotic effects. A common denominator in these cases is the control of tone by modulating type-A GABA receptors (Zhu et al., 2018).

### The pathophysiology of OSA

Some studies have sought to interpret OSA as a neuro-muscular respiratory disorder (Albdewi et al., 2018; Deak and Kirsch, 2014) caused by an alteration in the release of neurotransmitters, leading to the obstruction of the upper airways due to muscular hypotonia and insufficient dilatory muscle reactivation. The recurrent collapse of the pharyngeal muscles is aggravated by dysregulation of central neurotransmitters in the Central Nervous System and the Autonomic Nervous System, both sympathetic and parasympathetic.

**Figure 1:**
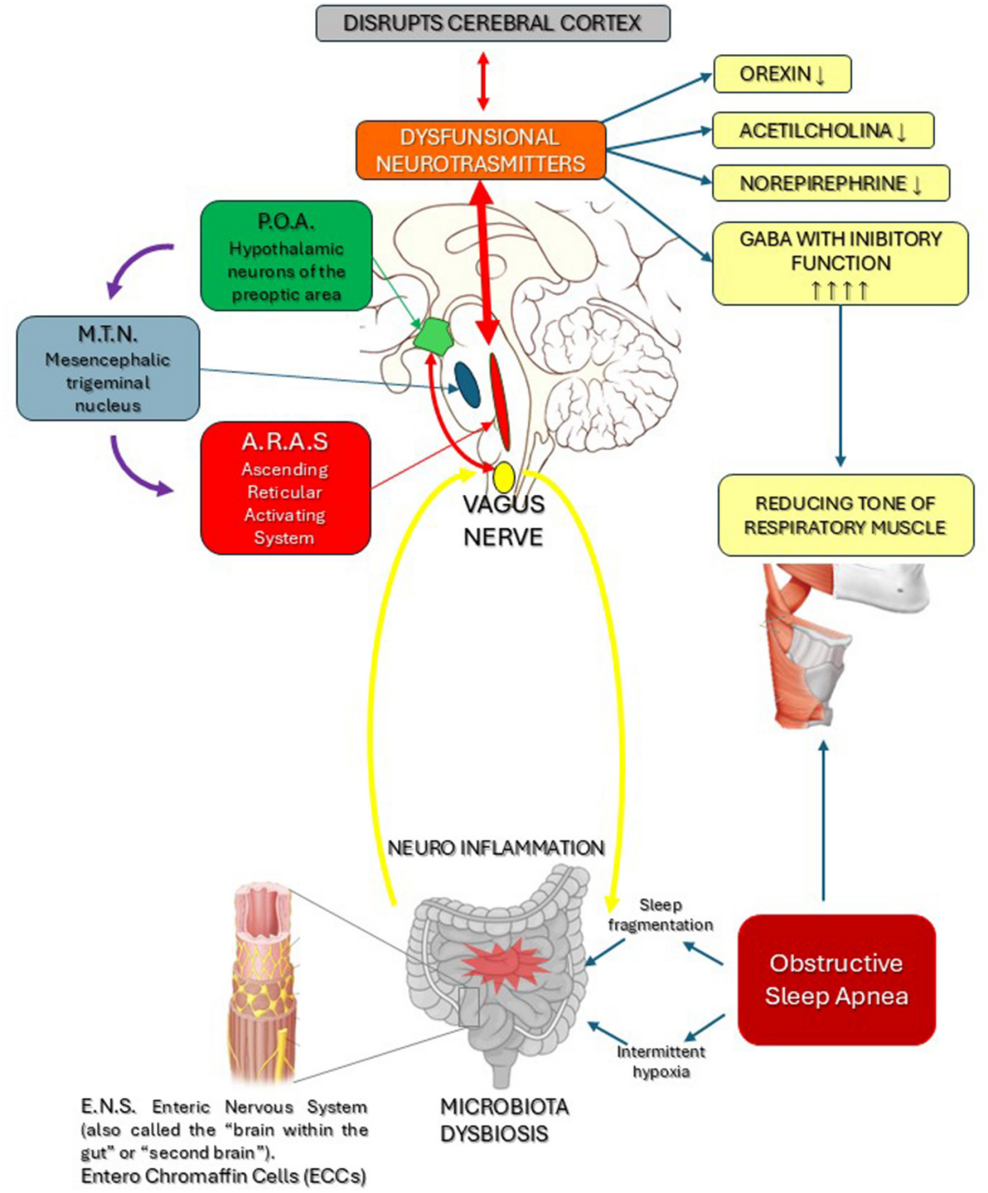
Dysbiosis determines, via the vagus nerve and the Enteric Nervous System (ENS), neuro-inflammation of the brainstem, which causes dysfunction of the neurotransmitters released by the POA and ARAS. This in turn disrupts the cerebral cortex and the respiratory cardiovascular systems, with reduced levels of Norepinephrine, Orexin, Dopamine and excessive GABA production, leading to hypotony in the muscles of the respiratory tract. Based on these findings, we conclude that a dysfunctional POA and MTN with excess GABAergic impulses reduces the sympathetic tone in the ARAS, with increased GABA-driven inhibitory activity in the respiratory muscles.

Another recent study (Andrisani and Andrisani, 2023) attributed a causal role in OSA to excessive nocturnal release of inhibitory neurotransmitters, such as GABA, by neurons in the hypothalamic region known as the Preoptic Area (POA). GABA reduces and slows down the activity of neurons in the Ascending Reticular Activating System (ARAS), which controls wakefulness by producing orexin, histamine, serotonin and noradrenaline. The inhibitory function of GABA is crucial for balancing the excitatory effect on muscle tone of other neurotransmitters including acetylcholine, dopamine and noradrenaline. Neurons in the Mesencephalic Trigeminal Nucleus (MTN) also participate in this neurophysiological process by producing the excitatory neurotransmitter glutamate, which in turn activates and positively stimulates the ARAS (Parrino et al., 2021; Allen et al., 2024; Sigel and Steinmann, 2012) (see Figure 1).

## Conclusion

On the basis of the data presented in the scientific literature examined here, the physio pathological mechanism hypothesized by us represents a new model of OSA pathogenesis that takes account of the complexity of the microbiota and of the above-described anatomo-functional and neuro-physio pathological interrelations.

In accordance with this model therefore, OSA can be explained by temporary nocturnal dysregulation of the activity of central neurotransmitters released by the ARAS. GABA has an inhibitory effect on the synthesis of noradrenaline, this effect being responsible for reducing the muscle tone of pharyngeal muscles. Reduced levels of norepinephrine released by the ARAS contribute to the development of nocturnal respiratory disorders. Reduced levels of orexin, norepinephrine, epinephrine, oxytocin, histamine (a crucial promoter of wakefulness that stimulates positive respiratory and cardiovascular responses) and testosterone (which in men stimulates the respiratory and cardiovascular systems) also play a role in this neuro-physio-pathological process.

Dysbiosis can cause an excess of POA-mediated inhibitory GABAergic impulses. This imbalance can in turn result in insufficient nocturnal activation by the MTN of central excitatory neurotransmitters such as glutamate (Glu), acetylcholine (Ach), histamine, dopamine (DA), norepinephrine (NE), and epinephrine (Epi), as well as neurohormones released by the hypothalamus such as oxytocin (Oxt). The resulting disturbance of nocturnal neurotransmitters can cause hypotonia of the hypopharyngeal and hypoglossal muscles and partial/total obstruction of the upper airway's characteristic of OSA during sleep. However, considerable progress in the study of GABA_A_ receptors notwithstanding, there is a great deal that remains unknown. We have yet to fully establish where they are present and to determine their molecular structure, and we do not have exhaustive knowledge of their many functions. The answers to these questions will not only provide information on the internal functioning of the nervous system, but could also facilitate the development of new targeted treatments.

Lastly, genetic engineering now holds the possibility of deploying a CRISPR-Cas system (Clustered Regularly-Interspaced Short Palindromic Repeats) that will enable modifications to the DNA of dysbiotic intestinal bacterial cells. This “*gene editing*” makes it possible to modify, remove or add specific DNA sequences, offering an innovative approach to the design of new gene therapies targeting the dysbiotic human microbiome and the regulation of central neurotransmitters. In the near future, big data generated by the omic sciences (genomics, proteomics, metabolomics, metagenomics, phenomics and transcriptomics) will enable a “tailored” approach to diagnosis and plausibly treatment.
